# Prevalence and gender distribution of the metabolic syndrome

**DOI:** 10.1186/1758-5996-2-1

**Published:** 2010-01-12

**Authors:** Anthonia O Ogbera

**Affiliations:** 1 Department of Medicine, Lagos State University Teaching Hospital, Ikeja, Lagos, Nigeria; 2 Department of Medicine, General Hospital Gbagada, Lagos, Nigeria

## Abstract

**Background:**

The Metabolic syndrome (MetS) is a cardiovascular risk factor of public health significance and of recent has become a topical issue. The prevalence of diabetes mellitus (DM) is on the increase and with this scenario, a possible increase in burden of DM which may be largely attributed to cardiovascular complications is expected. The objective of this report is to determine the prevalence of the MetS and compare gender characteristics in subjects with type 2 DM.

**Methods:**

Subjects with type 2 DM were recruited from an urban hospital for the study. Clinical data was obtained by interviewing the patients and referring to their Case folders. The anthropometric indices and blood pressure measurements were documented. Laboratory parameters analysed for included total cholesterol, high density and low density cholesterol, triglyceride and glycosylated haemoglobin. Statistical analysis included usage of Student's t test and chi square.

**Results:**

963 patients with type 2 DM aged between 35-85 years were recruited for the study. The main outcome measures included the prevalence of the metabolic syndrome and the gender differences of its components. The prevalence of the metabolic syndrome was 86%. The frequency of occurrence of the MetS was similar for men (83%) and women (86%) and increased with age in both sexes. The prevalence of MetS increased from 11% among participants aged 20 through 29 years to 89% in participants aged 70 through 79. In our patients with DM, the commonest occurring and least detected MetS defining parameters are central obesity and elevated triglyceride levels respectively. The components of the MetS that differed significantly in both sexes was HDL-C. The combination of the components of the MetS were comparable in both genders and 5.8% of the subjects with the MetS had all components of the MetS.

**Conclusion:**

The prevalence of the MetS in type 2DM is high in both genders and increases with age thus posing a potential high cardiovascular risk in this group of patients. The modifiable risk factors for the MetS should be a focus point in the management of subjects with type 2 DM,

## Introduction

The metabolic syndrome (MetS) is a cluster of cardiovascular risk factors that is characterized by obesity, central obesity, insulin resistance, atherogenic dyslipidemia, and hypertension[1]. Although there are different definitions of the metabolic syndrome, the uniform pathophysiology of this syndrome is insulin resistance[2]. A prominent clinical feature of this syndrome is abdominal or central obesity. The criteria [3] for the MetS include five variables namely, abdominal obesity, raised triglycerides, low high density alcohol (HDL), elevated blood pressure and a history of diabetes mellitus or impaired fasting glucose state. The importance of the metabolic syndrome lies largely in the development of cardiovascular diseases and type 2 diabetes mellitus[4].

The prevalence of metabolic syndrome increases with increasing glucose intolerance [5] and with the increasing world wide prevalence of DM, the expected increase in the frequency of occurrence of the MetS will expectedly be in geometric proportions. The prevalence of the MetS in the general population is estimated to be between 17-25% [6,7] and in people with DM, reported prevalence rates range from 59% to 61% [6-8].

In Nigeria, DM is an emerging non-communicable health problem as its prevalence is on the increase. The reported prevalence rates of MetS in Nigerians with and without DM are 22% and 59% respectively.

Published reports differ in the gender distribution of the MetS. Whilst some researchers report a higher incidence of the MetS in men [9,10] than women, the reverse is the case in some other reports[11]. A consistent finding in the prevalence of the metabolic syndrome, is age dependence- the prevalence of the MetS is often noted to increase with age [9,12]. This report aims to determine the frequency of occurrence, correlates and gender distribution of the components of the MetS in Nigerians with DM.

## Methods

Subjects with type 2 DM were recruited from the General hospital Gbagada and the Lagos state University Teaching hospitals, Lagos for the study. This report is part of a recent study on lipid profile in DM by the author [13]. Details of DM including duration, treatment type, presence of comorbidities, smoking and alcohol histories were obtained by interviewing the study subjects and also from information in their case folders. Clinical examination entailed determining the body mass indices and waist circumferences.

Laboratory assessment included obtaining venous blood samples in a fasted state for the determination of components of the lipid panel (total cholesterol, high density cholesterol, low density cholesterol and triglyceride), blood glucose and glycosylated haemoglobin levels (HBA1c). Serum glucose was measured using glucose-oxidase method and lipid profile by enzymatic-colorimetric method. Ethical approval was obtained from the Ethical committee of both hospitals and informed consent was obtained from the study subjects.

The presence of the metabolic syndrome was determined using the new definition[3]. The presence of three or more of any of the following is a pointer to the MetS. waist circumference (WC) greater than 102 cm in men and 88 cm in women; serum triglycerides (TG) level of at least 150 mg/dL (1.69 mmol/L); high-density lipoprotein cholesterol (HDL-C) level of less than 40 mg/dL (1.04 mmol/L) in men and 50 mg/dL (1.29 mmol/L) in women; blood pressure of at least 130/85 mm Hg; or serum glucose level of at least 100 mg/dL (5.6 mmol/L).

## Results

A total of 973 patients with diabetes mellitus were recruited for the study. The females and males were 703 and 260 in number respectively.

### Clinical characteristics of the study subjects

The mean age of the study subjects was 58.6 (10.5) years and the female:male ratio was 3:1. Smoking and alcohol histories were documented in 83 (9%) and 208 (21%) of the subjects. Hypertension was a notable feature which was present in 572 (59%) of the subjects. A summary of the clinical parameters of the study subjects is shown in Table 1:

**Table 1:** Clinical characteristics of the study subjects

Variable	Mean (SD*)	Range
Age (years)	58.7(9.9)	35-85
BMI(kg/m^2^)	28.8(5.9)	14.8-56.9
WC(cm)	94.8 (13.4)	32-165
Duration of DM (years)	6.9(6.6)	0.1-40
HBA1c	6.6(2.4)	3.6-18

*-Standard deviation

A comparison of the clinical and biochemical parameters of both sexes showed that the mean BMI and duration of DM of the female subjects were significantly higher than those of the males (29.5 kg/m^2 ^vs 27 kg/m^2 ^p = 0.0001, 7 vs 6.5, p = 0.03). The mean HBA1c, Low density lipoprotein (LDL-C) and total cholesterol (TCHOL) were also significantly higher in females than males respectively (6.7% vs 6.2% p = 0.015, 132.1 mg% vs117.4 mg% p = 0.02, 192 vs 183 p = 0.001).

Well over half-793- of the subjects (81%) were on oral hypoglycaemic agents, 64 (7%) were on insulin and 108 (11%) and 9(1%) respectively were on combination of insulin and OHA and sole dietary management respectively.

### The metabolic syndrome and clustering of its components

The total number of the subjects with the metabolic syndrome was 834 thus giving a prevalence rate of 86%. The most prevalent risk factor was abdominal obesity. The proportions of the defining parameters of MetS are shown in Table 2

**Table 2:** Frequency of occurrence of the components of the metabolic syndrome

MetS defining parameters	Frequency (%)
Waist circumference component	658 (80%%)
History of hypertension	562(67%)
Elevated Triglyceride	194 (23%)
Reduced high density lipoprotein cholesterol	576 (69%)

**Figure 1:**
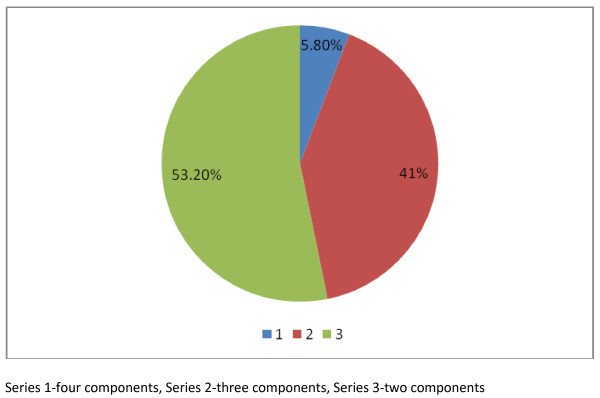
**Distribution of the components of the Mets**.

The distribution of the components of the metabolic syndrome is depicted in Figure 1:

For two components, the most frequent combinations were central obesity and reduced HDL-C (51.6% in women and 57.5% in men), for three components, the most frequent combinations were central obesity, hypertension and reduced HDL- (42.2% in women and 37.8% in men) and for four components (6.2% in women and 4.5% in men).

The proportion of the males with the MetS was comparable to that of the females with the MetS (83% vs 87%, p-0.2) The mean duration of DM was comparable in both sexes (7.1(6.2) vs 6.5(6.6) p-0.1). The overall prevalence of hypertension was 67% and the proportion of females with it was significantly higher than that of the males. (70% vs 605, p-0.04). Smoking and alcohol histories were documented more in males than females and these differences were statistically significant (for smoking 11 (2%) vs 60 (27%) p 0.0000001. alcohol history (71 (12%) vs 105 (47%) p-0.00001)

### Pattern of lipid subjects with the metabolic syndrome

**Figure 2:**
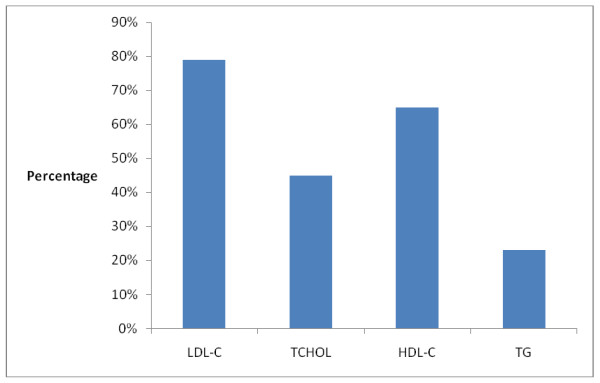
**Pattern of lipid abnormalities in subjects with the Mets**.

The pattern of lipid abnormalities showed that elevated LDL-C and reduced HDL-C were the commonly documented lipid abnormalities. This is shown in Figure 2.

The frequency of occurrence prevalence of the metabolic syndrome increased with age. The proportions of subjects with the metabolic syndrome in each age class is shown in Table 3

**Table 3:** Distribution of the metabolic syndrome by age classes

Age classes	All, N (freq)	MetS, N (freq)
20-29.9	9 (1%)	1(11%)
30-39.9	39 (4%)	28(72%)
40-49.9	118(12.1%)	97(82%)
50-59.9	271(27.9%)	238(88%)
60-69.9	405(41.6%)	353(87%)
70-79.9	123(12.6%)	110 (89%)
>80	8(0.8%)	7 (87%)

**Figure 3:**
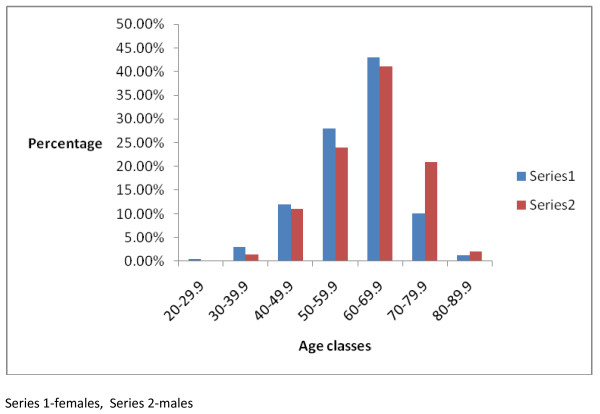
**Age-specific gender distribution of the prevalence of the Mets**.

The age specific distribution of the MetS was comparable in both genders except in ages 70 through 79.9. These results are shown in Figure 3.

The proportion of women with the MetS who had elevated blood pressure was significantly higher than men who had same (431 (76% vs 135 (61%), p = 0.01). Females with the metabolic syndrome tended to have higher total cholesterol and LDL-cholesterol than the males with the metabolic syndrome. A comparison of other clinical and biochemical parameters in both genders with the MetS is shown in Table 4

**Table 4:** Gender differences in clinical and biochemical parameters of subjects with the MetS

Variable	Males:	Females_	_p value_
WC(cm)	95.2 (11.9)	96.8	0.1
AGE (years)	60.8(10)	58.7(9.5)	0.05
BMI(kg/m^2^)	27.6(4.3)	30 (3)	0.0001
TG(mg%)	105.3 (55)	104.8 (5)	0. 8
CHOL(mg%)	185(43.2)	197.3(46.8)	0.001
LDL-C (mg%)	121(41.6)	133.9(49.9)	0.001
HDL-C (mg%)	40.1(16.5)	43.8 (19)	0.01

Quantitative data are displayed as mean values and standard deviation

### Comparison of clinical and biochemical between subjects with and without Mets

The subjects with the metabolic syndrome tended to be older and their mean LDL-C was higher than that of the subjects without the metabolic syndrome. The clinical and biochemical parameters are shown in Table 5.

**Table 5:** Clinical and biochemical parameters of subjects with the Mets and without the Mets.

Variable	MetS	Non MetS_____	p value_______
TCHOL(mg%)	193.8(46)	193.7 (48.8)	0.9
LDL-C(mg%)	130.3 (48)	114 (50)	0.001
Age (years)	59.2 (9.9)	55.1 (13.7)	0.001
Smoking	71 (8.5%)	12 (8.7%)	0.9
Alcohol	126 (21%)	32 (23%)	0.1
HBA1c(%)	6.6 (2.4)	7(2.3)	0.08
BMI(kg/m^2^)	29.3(29.30	25.8(5.9)	0.00001
Duration of DM (years)	6.9 (6.5)	7.2(7)	0.3

Quantitative data are displayed as mean values and standard deviation

## Discussion

The MetS is a cluster of cardiovascular risk factors including obesity, hypertension and dyslipidaemia that increases the risk of the development of type 2 diabetes mellitus and cardiovascular disease. The risk factors of MetS include obesity, aging, sedentary lifestyle, diabetes mellitus, coronary heart disease and lipodystrophy [14,15]. It is estimated that t a large majority of patients with type 2 DM or impaired glucose tolerance have the metabolic syndrome[8]. I note similar findings in this study and report the prevalence rate of Mets to be 86%. The prevalence rates of the Mets in both genders as noted in this report are comparable to that of a previous report on the Mets[16]. The role of age as a risk factor of MetS cannot be overemphasized as age dependency of the syndrome's prevalence is seen in most populations around the world[6]. In this report, the prevalence of MetS is noted to increase from 11% among participants aged 20 through 29 years to 89% in participants aged 70 through 79. Although this study showed that the mean age of men with MetS was significantly higher than that of women, the age specific prevalence of MetS however was similar in both genders except for ages 70-79 where the proportion of men with MetS was found to be almost twice that of females. These findings are contrary to those documented in a Seychelles'[17] population where the greatest prevalence of MetS using the ATP definition was highest at age 45-54 for men. In a Finnish [18] study on Mets, the prevalence of the MetS was found to increase with increasing age in women.

There are currently two major definitions for metabolic syndrome and these are provided by the International Diabetes Federation[16] and the revised National Cholesterol Education Program [19], respectively. In a bid to harmonize the different criteria for the definition of the Mets, several bodies have met and issued a joint statement making some alterations in the International Diabetes Federation (IDF) definition of the Mets. The main difference concerns the measure for central obesity which was proposed should no longer be an obligatory component for the diagnosis of the Mets although the waist measurement would continue to be a useful preliminary screening tool. The proposed blood glucose criterion as one of the defining parameters of the Mets is now 100 mg% or more[3].

The components of the metabolic syndrome vary in their rates of occurrence. The Seychelles[16] study reported high blood pressure and adiposity as the MetS defining criteria that occurred most commonly irrespective of the MetS definitions used. I however report central obesity and reduced HDL-cholesterol as the prevalent components of the Mets in our study subjects. The mean waist circumferences were comparable in both sexes with Mets but significantly lower HDL levels were noted in men than in women. Central obesity was found in 80% of the study subjects. This finding is not surprising given the observation that central obesity plays a central role in the development of the MetS and appears to precede the appearance of the other MetS components[20]. It is however pertinent to note that although the specific role of central obesity in patients with the metabolic syndrome remains unexplained, active brown adipocytes which accumulate in central locations have been found to be metabolically active. In addition, many studies have confirmed the existence of a tighter correlation of central obesity with insulin resistance, dyslipidemia, hypertension, and atherosclerotic heart disease than for obesity without regard to pattern [21]. Some researchers have report lower rates-25%- of occurrence of central obesity in the MetS[22].

The pattern of lipid abnormalities in this study was such that LDL-C although not a component of MetS was the commonest documented lipid abnormality in subjects with the MetS. The occurrence of elevated LDL-C in people with the Mets has been noted to increase the magnitude of the risk for developing coronary artery disease[23]. I report a gender difference in the pattern of lipid abnormality in subjects with the Mets. LDL-C and TCHOL were higher in women than in men with the MetS. The men with the MetS however had higher mean TG and lower HDL-C values than women with the MetS. This pattern of lipid abnormality was documented in a recent Nigerian study [7].

Gender differences were also documented in the occurrence of hypertension in the metabolic syndrome. We report a prevalence rate of hypertension of 67%. This is similar to reports to reports from the Middle East[22] and Nigerian reports[7]. There were however significant gender differences in hypertension and females were found to have a significantly higher incidence rates of hypertension than men.

Although each of the components of the metabolic syndrome individually have been identified as risk factors for cardiovascular disease, an individual with three or more components is at particularly high risk. I report a comparable distribution of the components of the MetS in both sexes. A small proportion-5.8% -of our subjects with type 2 DM have all the components of the MetS. This is in contradistinction to the report by Fezeu et al[9] who reported the absence of a combination of four component of the MetS in their study subjects. Potential factors that may have accounted in the gender differences in the distribution of the components of the MetS include an older age in men and significant smoking and alcohol histories.

A comparison of biochemical parameters other than those that define the MetS in both sexes showed that subjects with the MetS were significantly older, had higher body mass indices and higher low density cholesterol than those without the MetS.

## Conclusion

This study has shown the unacceptably high prevalence rate of MetS in both sexes with type 2 DM thus predicting a high disease burden of type 2 DM from possible cardiovascular complications.

## Competing interests

The authors declare that they have no competing interests.

## Authors' contributions

AOO designed the study, participated in data collation, statistical analysis, funding and writing the draft of the manuscript
